# Supplementary material for: Omics Approaches in Adipose Tissue and Skeletal Muscle Addressing the Role of Extracellular Matrix in Obesity and Metabolic Dysfunction
Source: Int J Mol Sci. 2021 Mar 9;22(5):2756. doi: 10.3390/ijms22052756 (PMC7963192; doi:10.3390/ijms22052756)
Supplement: Supplementary file 1 [file ijms-22-02756-s001.pdf]

Figure S1. Study selection according to PRISMA 2009 Flow Diagram.

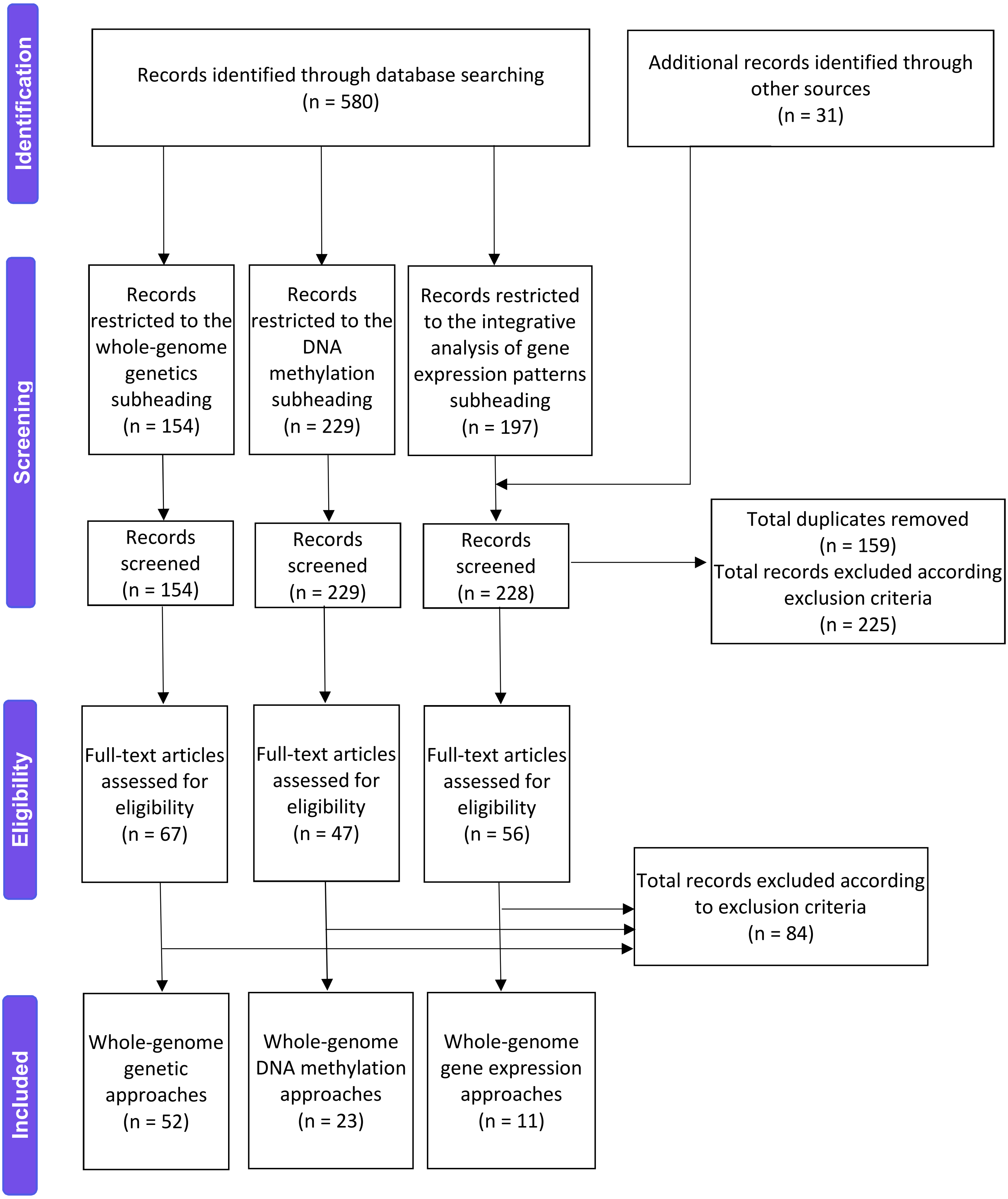

Table S1. Ontology terms selected for the search and number of genes annotated in each term

Term Type	Term description	Nº of annotated genes
GO TERMS	Cell adhesion molecule binding	541
	Regulation of cell adhesion	431
	Collagen containing extracellular matrix	351
	Cell matrix adhesion	159
	Extracellular matrix organization	88
	Positive regulation of endothelial cell migration	85
	Negative regulation of endothelial cell migration	74
	Extracellular matrix	66
	Extracellular matrix disassembly	34
	Extracellular matrix assembly	18
	Extracellular matrix structural constituent	18
	Extracellular matrix disassembly	15
	Costamere	12
	Matrix metallopeptidase secretion	9
	Extracellular matrix constituent secretion	8
	Dystrophin associated glycoprotein complex	7
	Substrate dependent cell migration	7
	Cell matrix adhesion	6
	Cell adhesion involved in heart morphogenesis	5
	Dystroglycan complex	5
	Extracellular matrix binding	5
	Extracellular matrix binding	5
	Regulation of endothelial cell migration	5
	Extracellular matrix cell signaling	3
	Extracellular matrix organization	3
	Cell adhesion involved in sprouting angiogenesis	2
	Extracellular matrix protein binding	2
	Transforming growth factor beta activation	2
	Integrin complex	1
	Positive regulation of collagen catabolic process	1
	Regulation of cell migration involved in sprouting Angiogenesis	1
KEGG terms	Regulation of actin cytoskeleton	84
	Focal adhesion	64
	Tight junction	38
	Adherents junction	23
	ECM-receptor interaction	8

Abbreviations: GO, Gene Ontology; KEGG, Kyoto Encyclopaedia of Genes and Genomes; ECM, extracellular matrix

Table S2. Boolean search equations employed.

Approaches	Search syntax
Whole-genome genetics	*(GENE_SYMBOL(i)[Title/Abstract]) OR (GENE_SYMBOL(i)[MeSH Terms]) AND (human? OR children OR teenage? OR adolescent OR adult?) AND (obesity[Title/Abstract]) AND (SNPs*) AND ((GWAS[Title/Abstract]) OR (genome wide association[Title/Abstract]))*
Whole-genome DNA Methylation	*(GENE_SYMBOL(i)[Title/Abstract]) OR (GENE_SYMBOL(i)[MeSH Terms]) AND (human? OR children OR teenage? OR adolescent OR adult?) AND (obesity) AND (*methylation*) AND ((adipose*[Title/Abstract]) OR (blood[Title/Abstract]) OR (*muscle*[Title/Abstract]))*
Whole-genome gene expression	*(GENE_SYMBOL(i)[Title/Abstract]) OR (GENE_SYMBOL(i)[MeSH Terms]) AND (humans) AND (obesity) AND (gene expression) AND ((extracellular matrix[MeSH Terms]) OR (extracellular matrix proteins[MeSH Terms])) AND ((adipose tissue) OR (muscle, skeletal))*

The database consulted was NCBI/PudMed. The iterative search was performed by replacing *GENE_SYMBOL(i)* for the corresponding *i*th gene from the 2186 selected genes list.
